# Dispersion and Lubrication of Zinc Stearate in Polypropylene/Sodium 4-[(4-chlorobenzoyl) amino] Benzoate Nucleating Agent Composite

**DOI:** 10.3390/polym16131942

**Published:** 2024-07-07

**Authors:** Yapeng Dong, Fuhua Lin, Tianjiao Zhao, Meizhen Wang, Dingyi Ning, Xinyu Hao, Yanli Zhang, Dan Zhou, Yuying Zhao, Xinde Chen, Bo Wang

**Affiliations:** 1 School of Chemical Engineering and Technology, Taiyuan University of Science and Technology, Taiyuan 030024, China; s202221211035@stu.tyust.edu.cn (Y.D.); s202221211044@stu.tyust.edu.cn (T.Z.); s202321111073@stu.tyust.edu.cn (M.W.); 202021070217@stu.tyust.edu.cn (D.N.); 201921050134@stu.tyust.edu.cn (X.H.); 2021051@tyust.edu.cn (Y.Z.); 2021053@tyust.edu.cn (D.Z.); yuyingzhao@tyust.edu.cn (Y.Z.); 2 School of Traffic Engineering, Shanxi Vocational University of Engineering Science and Technology, Jinzhong 030619, China; linfuhua@sxgkd.edu.cn; 3 Key Laboratory of Renewable Energy, Guangzhou Institute of Energy Conversion, Chinese Academy of Sciences, Guangzhou 510640, China

**Keywords:** sodium 4-[(4-chlorobenzoyl) amino] benzoate, zinc stearate, composite nucleating agent, polypropylene

## Abstract

Zinc stearate (Znst) was physically blended with the sodium 4-[(4 chlorobenzoyl) amino] benzoate (SCAB) to obtain the SCAB-Znst composite nucleating agent. Znst was used to improve the dispersion property of SCAB and exert a lubricating effect on the PP matrix. The scanning electron microscopy and the fracture surface morphology of the PP/SCAB composite illustrated that the addition of Znst greatly reduced the aggregation phenomenon of SCAB in the PP matrix. The result of the rotary rheometer indicated that Znst exhibits internal lubrication in PP. The DSC result illustrated that the crystallization properties of PP were improved. Compared with pure PP, the *T_c_* of the PP/SCAB composite increased by 1.44 °C (PP/Znst), 13.48 °C (PP/SCAB), and 14.96 °C (PP/SCAB-Znst), respectively. The flexural strength of pure PP, PP/SCAB, and PP/SCAB-Znst were 35.8 MPa, 38.8 MPa, and 40.6 MPa, respectively. The tensile strength of the PP/SCAB and PP/SCAB-Znst reached the values of 39.8 MPa and 42.9 MPa, respectively, compared with pure PP (34.1 MPa). The results demonstrated that Znst can promote the dispersion of SCAB in the PP matrix while exerting a lubricating effect, which enabled the enhancement of the crystalline and mechanical properties of PP.

## 1. Introduction

Polypropylene (PP) has excellent properties, such as low cost, easy processing, and high heat resistance, and it is widely used in food packaging, automotive, and other industrial production fields [1,2,3]. However, the low crystallization rate and the poor mechanical properties limit the application of PP [4,5]. At present, blending with the nucleating agent can greatly accelerate the crystallization rate of PP through heterogeneous nucleation [6]. Meanwhile, the nucleating agent can also change the crystal structure of PP to improve the mechanical properties of PP [7].

The nucleating agent used in the modification of PP can be categorized into organic and inorganic. Compared with the inorganic nucleating agent, the organic nucleating agent has the advantages of high nucleation efficiency and good interface compatibility, which is a commonly used additive in the high-value modification of PP [8]. Currently, the commercialized organic nucleating agents include the calcium salt of hexahydrophthalic acid (HPN-20E) [9], 2,2-methylene-bis (4, 6-di-tert-butylphenyl) phosphate hydroxyl aluminum (NA-21) [10], bicyclo[2.2.1]heptane-2,3-dicarboxylic acid disodium salt (HPN-68L) [11], 1,3;2,4-bis(3,4-dimethylbenzylidene)sorbitol (Millad 3988) [12], and N,N′-dicyclohexyltereph-th alamide (TMB-5) [13]. The organic nucleating agent displays superior dispersion properties within the PP matrix when compared to the inorganic nucleating agent. However, the dispersion property of the organic nucleating agent in the PP matrix still needs improvement because the organic nucleating agents are generally small molecular compounds.

Xie et al. [14] prepared a self-dispersed calcium pimelate organic nucleating agent via in situ ion exchange with succinic acid and calcium stearate. The dispersion of the calcium pimelate organic nucleating agent in the iPP matrix was improved due to the presence of stearic acid segments. Compared with pure iPP, the crystallization temperature (*T_c_*) of the iPP/nucleating agent blend increased by 4–5 °C. The impact strength of the iPP/nucleating agent blend increased by 52.1%. Lin et al. [15] used [2,2′-methylene-bis(4,6-di-tert-butylphenyl) phosphate] nucleating agent (APAl-OH) physical blending with lithium laurate (12Li) to improve the dispersion of APAl-OH in the PP matrix. The results showed that the dispersion of APAl-OH in the PP matrix was significantly improved. Therefore, the crystallization rate, the flexural modulus and impact strength were greatly increased. He et al. [16] investigated the effect of montmorillonite on the nucleation efficiency of the TMB-5 nucleating agent. The results showed that the arrangement of the TMB-5 on the surface of montmorillonite forms a self-assembled structure caused by the hydrogen bonding between montmorillonite and TMB-5, which can improve the dispersibility of the TMB-5. Thus, the nucleation efficiency of the iPP was significantly improved. Obviously, the organic nucleating agent’s physical blending with the long-chain fatty acid salt can improve the dispersion property of the organic nucleating agent in the PP matrix, which has the advantages of being effective and easily operated. Moreover, the addition of long carbon chains in the polymer matrix can play a role in internal lubrication, which can improve crystallization and mechanical properties [17].

Mao et al. [18] prepared MAFT by grafting Fischer–Tropsch wax (FTW) with the maleic anhydride, and MAFT was reacted with ATP to obtain FATP. FATP was used to modify the isotactic polybutene (iPB). The results showed that the crystallization rate constant and the flexural strength of iPB+5% FATP reached 0.043 and 12.45 MPa at the same annealing time. The increase was attributed to the long carbon chains in MAFT, which improved the interfacial compatibility between ATP and iPB and accelerated the alignment of the molecular chains and the transformation of the crystals in the iPB. Wu et al. [19] used calcium stearate (Cast), zinc stearate (Znst), and aluminum stearate (Alst) physical blending with sorbitol nucleating agent. The results indicate that the addition of Cast, Znst, and Alst can enhance the dispersion of sorbitol nucleating agents in the PP matrix. Meanwhile, Cast, Znst, and Alst act as an internal lubricant and accelerate the rearrangement of molecular chains, which can improve the comprehensive properties of PP. Balkaev et al. [20] combined the dicalcium salt of 1-hydroxyethane-1, 1-diphosphonic acid (HEDP) with Znst through physical blending to obtain HEDP/Znst. The result showed that the addition of HEPD/Znst increased the crystallinity and flexural modulus of the iPP by 4% and 8%, respectively, which indicates that Znst can promote the dispersion of HEDP and plays a lubricating role in the iPP matrix.

Milliken & Company has developed a kind of organic nucleating agent, HPN-210M, for the modification of polyethylene (PE). The principal component of the HPN-210M containing organic nucleating agent is sodium 4-[(4-chlorobenzoyl) amino] benzoate (SCAB). The research showed that the addition of SCAB can accelerate the crystallization rate of the HDPE by 5.2% and remarkably improve the mechanical properties of the HDPE [21]. For this reason, SCAB has great potential for use in the PP modification. As a small molecular compound, the defect of poor dispersion property in the PP matrix of SCAB still exists, which can significantly reduce the modification effects of SCAB. PP and PE have similar molecular structures, and SCAB has the potential to improve the crystallization behavior of PP. However, SCAB with small molecule compounds may cause the problem of poor dispersion in the PP matrix.

In this study, in order to improve the dispersion of organic nucleating agents in the PP matrix and provide other effects, SCAB was physically blended with Znst to obtain a SCAB-Znst composite nucleating agent. The molecular structure of SCAB-Znst before and after physical blending was investigated using Fourier transform infrared (FT-IR). The microstructure and the thermal stability of the SCAB-Znst composite nucleating agent were observed with scanning electron microscopy (SEM) and a thermogravimetric analyzer (TGA), respectively. The PP/SCAB composite was prepared using the melt blending method. The non-isothermal crystallization behavior of the PP/SCAB composite was investigated with a differential scanning calorimeter (DSC), and the non-isothermal crystallization kinetics of the PP/SCAB composite were calculated using the Jeziorny method. The crystal structure was observed using a polarizing microscope (POM). The dispersion property of SCAB in the PP matrix was observed using SEM. The rheological behavior of the PP/SCAB composite was used to describe the lubrication effect of Znst. The mechanical properties of the PP/SCAB composite were finally evaluated and discussed.

## 2. Materials and Methods

### 2.1. Materials

PP (045) with a melt flow index of 3.5 g/10 min was supplied by Shandong Orient Hongye Chemical Co., Ltd. (Shouguang China). SCAB was purchased from Shanxi Advance Science Green Industry Research Institute Co., Ltd. (Taiyuan China). Znst was purchased by Shanghai Macklin Biochemical Technology Co., Ltd. (Shanghai China).

### 2.2. Preparation of the SCAB-Znst Composite Nucleating Agent

SCAB and Znst with a mass ratio of 1:1 (*w*/*w*) were mixed in a high-speed mixer (SHR10L, Zhangjiagang Jainuo Machinery Co., Ltd., Zhangjiagang China) at a rotational speed of 800 r/min for 2 min to obtain SCAB-Znst.

### 2.3. Characterization of SCAB-Znst Composite Nucleating Agent

The FT-IR analysis of the SCAB-Znst composite nucleating agent was performed with an FT-IR spectrometer (iS10, Thermo Scientific Company, Waltham, MA, USA) using 64 scans per sample.

The microstructure of the SCAB-Znst composite nucleating agent and the fracture surface of the PP/SCAB composite were characterized by the SEM (Quanta 250 FEG, FEI, Hillsboro, OR, USA) at an accelerated voltage of 10 kV.

The thermogravimetry (TG) analysis was performed on a TGA (TGA-1, Mettle Toledo Company, Zurich, Switzerland). The samples were heated from 30 °C to 600 °C at a heating rate of 10 °C/min under a N_2_ atmosphere. The DTG curve was obtained by deriving TG curve data, which was used for data analysis.

### 2.4. Preparation of the PP/SCAB Composite

The formula of the PP/SCAB composite is shown in Table 1. Each component of the PP/SCAB composite was mixed in a high-speed mixer at a rotational speed of 25,000 r/min for 2 min. The evenly mixed samples were fed into the micro twin-screw extruder (WLG10A, Shanghai Xinshuo Precision Machinery Co., Ltd., Shanghai China) for extrusion to obtain the PP/SCAB composite, and the PP/SCAB composite was molded for standard test specimens using a microinjection machine (WZS10D, Shanghai Xinshuo Precision Machinery Co., Ltd., Shanghai China) with an injection pressure of 0.2 MPa at 200 °C.

### 2.5. Characterization of the PP/SCAB Composite

The non-isothermal crystallization behavior of the PP/SCAB composite was analyzed using DSC (Q1000, TA company, New Castle, DE, USA). The samples were cooled from 220 °C to 30 °C at different speeds (5, 10, 20 °C /min), and then heated from 40 °C to 220 °C at 10 °C/min. Crystallization and melting processes were recorded for subsequent data analysis.

The POM observations of the PP/SCAB composite were carried out using a polarized optical microscope (DM2700, Leica, Wetzlar, Germany) and combined with a hot stage (THMS 600, Linkam, UK) to control the temperature. The temperature program was set as follows: the PP/SCAB composite was first heated to 210 °C at 10 °C/min for 5 min, then cooled to 135 °C at 40 °C/min and held at 135 °C for 30 min.

The rheological properties of the PP/SCAB composite were determined using a rotational rheometer (DHR-2, TA Company, New Castle, DE, USA) in the oscillatory mode. The measurements were performed in the dynamic mode and 25 mm parallel plate geometry with a gap setting of about 2 mm. The temperatures were 180 °C, 190 °C, 200 °C, and 210 °C, and the scanning frequency was 0.1 Hz.

The tensile strength of the PP/SCAB composite was assessed following the GB/T 1040-2006, utilizing a universal testing machine (TY-8000A, Jiangsu Tianyuan Test Instrument Co., Ltd., Yangzhou, China) at a speed of 5 mm/min. The flexural strength of the PP/SCAB composite was performed according to GB/T 9341-2008, utilizing the universal testing machine at a speed of 10 mm/min. Five samples were tested individually, and the average results were recorded.

## 3. Results and Discussion

### 3.1. FTIR Spectra of the SCAB-Znst Composite Nucleating Agent

**Figure 1:**
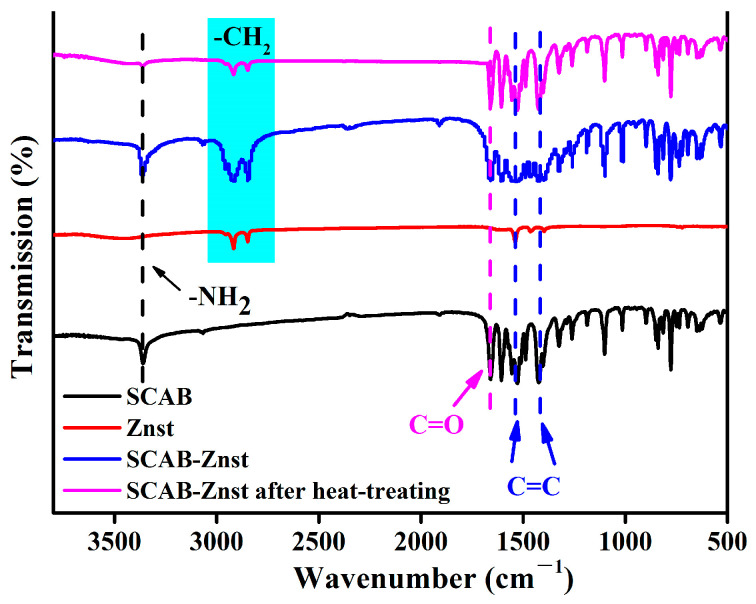
The FTIR spectra of the SCAB-Znst composite nucleating agent.

The FTIR spectra of the SCAB-Znst composite nucleating agent are shown in Figure 1. The characteristic absorption peaks at 3367 cm^−1^ and 1605 cm^−1^ are -NH_2_ and C=O of the SACB, respectively. The peaks of the C=C bond of the benzene ring in SCAB presented at 1525 cm^−1^ and 1417 cm^−1^. Meanwhile, the new characteristic peaks at 2921 cm^−1^ and 2844 cm^−1^ appeared in SCAB-Znst, which revealed the presence of symmetric and asymmetric stretching vibrations of -CH_2_ in Znst. The results demonstrated that SCAB and Znst had been successfully combined. Furthermore, the results indicated that Znst did not alter the chemical structure of SCAB. Concurrently, the changes in the chemical structure of SCAB-Znst at the PP processing temperature (200 °C) should be investigated further. SCAB-Znst was heat-treated in the micro twin-screw extruder at 200 °C for 5 min. The FTIR spectra of SCAB-Znst after heat-treating had no change, which indicates that the molecular structure of SCAB-Znst does not change during the processing of the PP/SCAB composite.

### 3.2. SEM Photograph of the SCAB-Znst Composite Nucleating Agent

**Figure 2:**
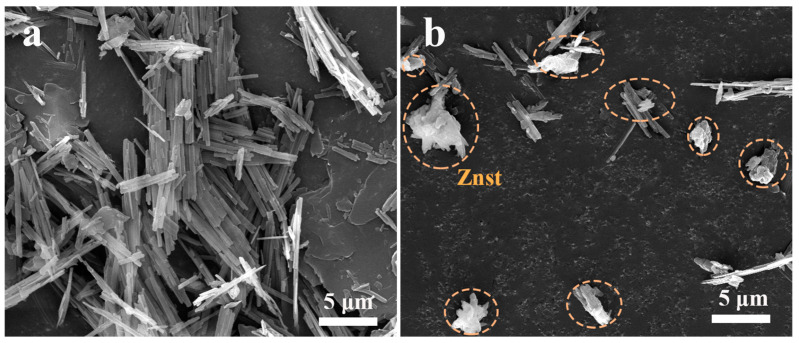
The SEM photograph of SCAB-Znst. (**a**) SCAB; (**b**) SCAB-Znst.

The microscopic morphology of the SCAB-Znst composite nucleating agent is shown in Figure 2. The results demonstrated that the microscopic morphology of SCAB exhibited slender strips, and the SCAB slender strips were tightly packed together. As shown in Figure 2b, the addition of Znst did not change the microscopic morphology of SCAB. Moreover, the aggregation of SCAB was obviously improved, which was presented as the slender strips exhibited a tendency to separate. This result may promote the uniform dispersion of SCAB in the PP matrix, thereby achieving better modification effects.

### 3.3. TG Analyses of the SCAB-Znst Composite Nucleating Agent

**Figure 3:**
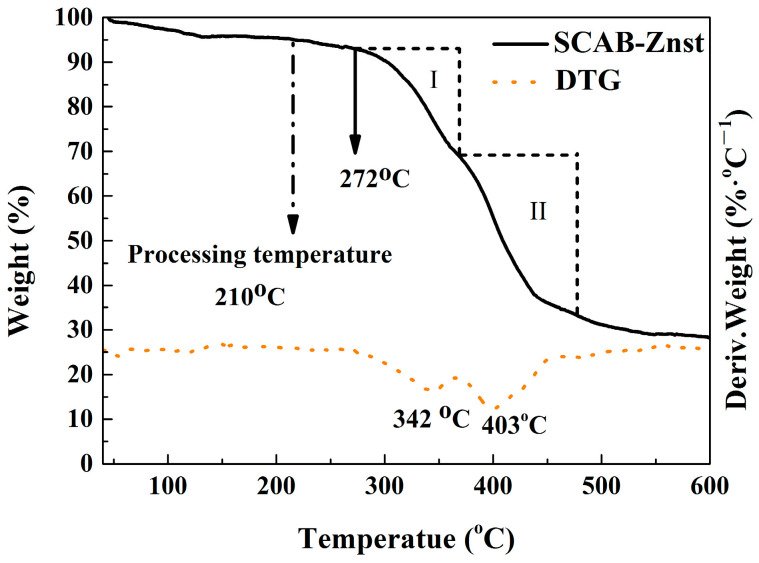
The TG and DTG curves of the SCAB-Znst composite nucleating agent.

Figure 3 shows that the initial decomposition temperature starts at around 272 °C. In the beginning, the quality of SCAB and Znst slightly decreased because of the removal of bound water of Znst at 100 °C–200 °C. In phase I, the weight of SCAB-Znst was gradually lost and was accompanied by a weak peak with a peak temperature of 342 °C, and the weight loss was approximately 30%. In phase II, there was a marked increase in mass loss, which was accompanied by the appearance of a distinct weight loss peak. The peak temperature was 403 °C, with a weight loss of about 39%. The maximum possible processing temperature of PP is 210 °C, while the minimum decomposition temperature of SCAB-Znst is 272 °C. Therefore, SCAB-Znst will not resolve during the processing, which can be used as a dispersive-type nucleating agent on the PP modification.

### 3.4. Non-Isothermal Crystallization Process of the PP/SCAB Composite

**Figure 4:**
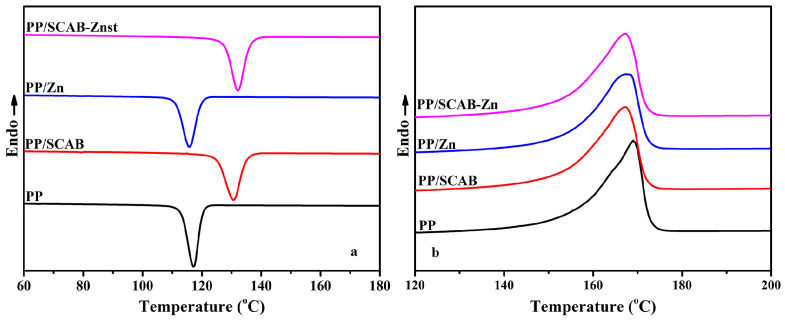
Non-isothermal crystallization DSC curves of the PP/SCAB composite (cooling rate 10 °C/min). (**a**) Non-isothermal crystallization curve; (**b**) Secondary melting curve.

Figure 4 presents the non-isothermal crystallization DSC curves of the PP/SCAB composite, and the crystallization peak parameters of the PP/SCAB composite are listed in Table 2. Compared with pure PP, the *T_c_* of the PP/SCAB composite increased by 1.44 °C (PP/Znst), 13.48 °C (PP/SCAB), and 14.96 °C (PP/SCAB-Znst), respectively. The order of crystallinity (*X_c_*) of the composites was PP < PP/Znst < PP/SCAB < PP/SCAB-Znst. The results indicate that SCAB can be used as an effective nucleating agent for PP, which can greatly improve the crystallization properties of PP. Moreover, the unique slender strip shape of SCAB provides favorable conditions for PP crystallization, which can increase the orderly structure of the crystallization process and the degree of crystal perfection [22].

After the physical blending of SCAB and Znst, the long carbon chain fatty acid salt Znst has an internal lubricating effect, which can promote the dispersion of SCAB in the PP matrix and further improve the crystallization properties of the PP. It is not difficult to see from Table 2 that the *T_c_* moved to the high-temperature region, and the melting temperature (*T_m_*) remained basically unchanged after adding SCAB-Znst.

### 3.5. Non-Isothermal Crystallization Kinetics Analysis of the PP/SCAB Composite

**Figure 5:**
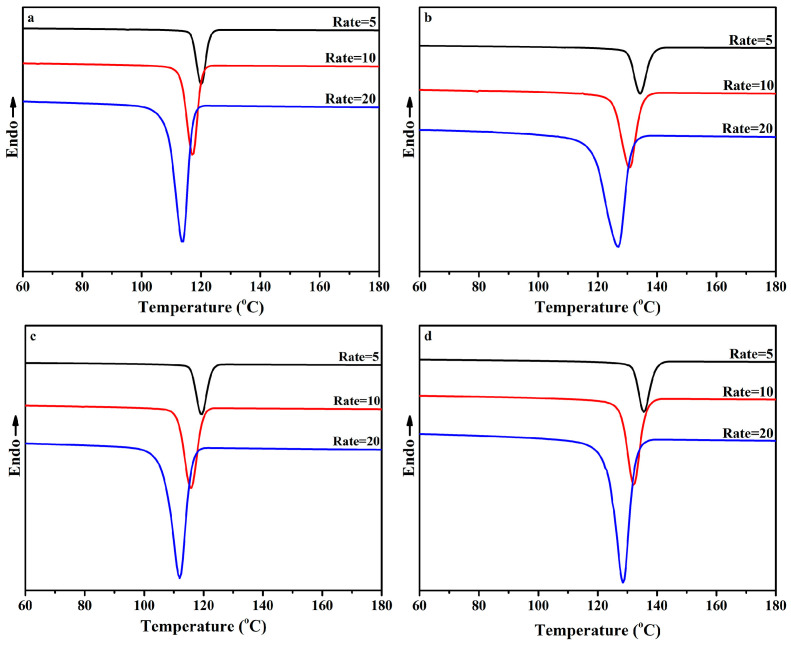
Non-isothermal DSC curves of the PP/SCAB composites at different cooling rates. (**a**) PP; (**b**) PP/SCAB; (**c**) PP/Znst; (**d**) PP/SCAB-Znst.

The non-isothermal crystallization curves of the PP/SCAB composite at three cooling rates of 5 °C/min, 10 °C/min, and 20 °C/min are shown in Figure 5. It can be seen that the crystallization peak of the PP/SCAB composite gradually widens with the increase in cooling rate, and the *T_c_* moves toward a low temperature. The primary cause of the phenomenon is the restricted molecular chain movement at lower temperatures, which results in the formation of crystal defects [23].

**Figure 6:**
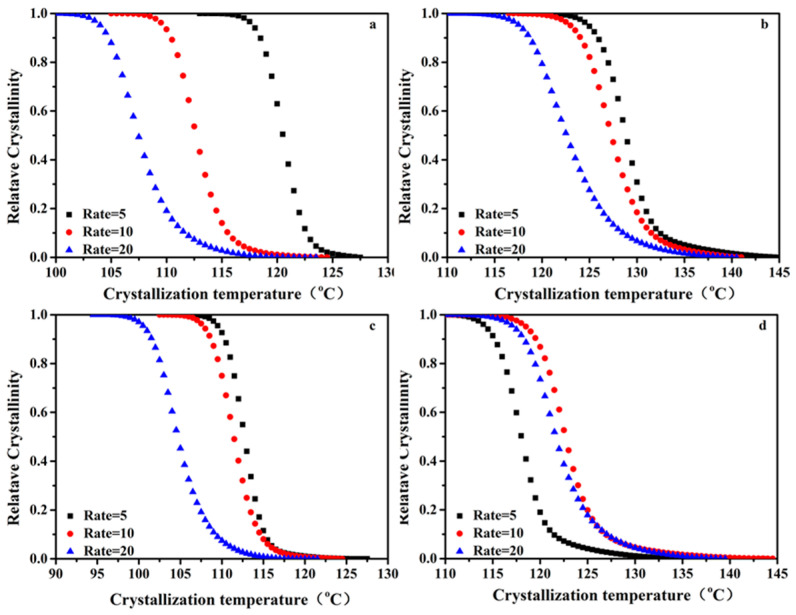
Relationship between relative crystallization and crystallization temperature of the PP/SCAB composite during non-isothermal crystallization. (**a**) PP; (**b**) PP/SCAB; (**c**) PP/Znst; (**d**) PP/SCAB-Znst.

In order to further analyze the non-isothermal crystallization of the PP/SCAB composites, the non-isothermal crystallization kinetics of the PP/SCAB composite were compared. Based on the assumption that the evolution of crystallinity is linearly proportional to the evolution of heat released during crystallization, the relative crystallinity *X_w_*(*t*) was calculated by integrating the exothermic peak with Equation (1).

(1) $$X_{w}\left(t\right)=\int_{t_{0}}^{t}(\frac{\mathrm{dH}}{\mathrm{dt}})\mathrm{dt}/\int_{t_{0}}^{t_{\infty }}(\frac{\mathrm{dH}}{\mathrm{dt}})\mathrm{dt},$$

where *dH/dt* is the crystallization heat flow rate at a temperature of *t*, *t*_0_ indicates the temperature at which crystallization begins, and *t_∞_* is the temperature at which crystallization completes. In Equation (1), *X_w_*(*t*) at any crystallization temperature is converted to the volume fraction relative crystallinity *X_v_*(*t*); the relationship between relative crystallinity and the crystallization temperature of the PP/SCAB composite during non-isothermal crystallization was obtained, as shown in Figure 6.

**Figure 7:**
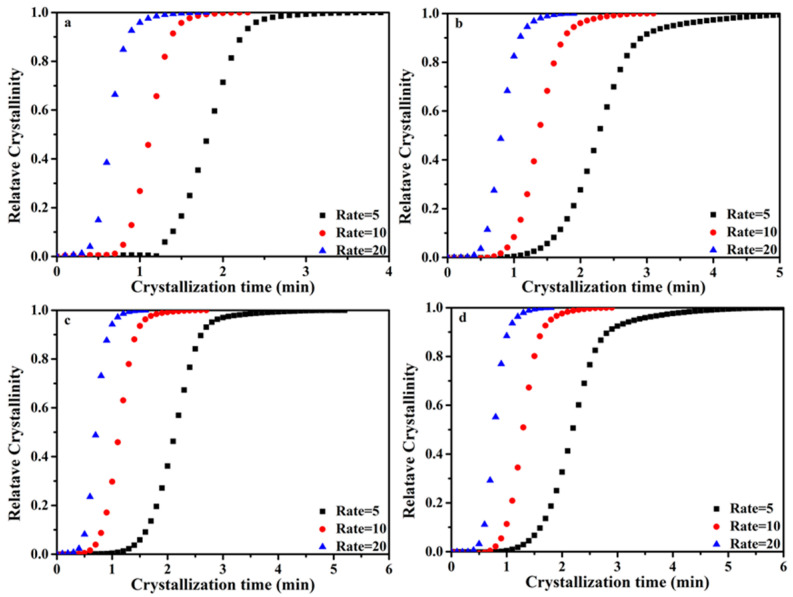
Relationship between relative crystallization and crystallization time of the PP/SCAB composite during non-isothermal crystallization. (**a**) PP; (**b**) PP/SCAB; (**c**) PP/Znst; (**d**) PP/SCAB-Znst.

The relationship between crystallinity *X*(*t*) and temperature *T* can be converted to the relationship between crystallinity *X*(*t*) and time t using Equation (2), where *T* is the temperature at time *t* and *φ* is the cooling rate, as shown in Figure 7.

t = (T_0_ − T)/φ (2)

The Avrami equation (Equation (3)) is generally used to describe the isothermal crystallization kinetics of crystalline polymers. The equation describes how a solid changes from one substance or phase state to another at a constant temperature. It can also describe certain types of chemical reactions [24].

(3) $$\log[-\ln(1-\;X_{v}(t))]=n\;\log\;t+\log\;K$$

*log Kc* = (*log K*)/*φ* (4)

In Equation (3), *n* is the Avrami index, and the size of the n value usually represents the crystal growth mode, and the value of *n* ranges from 1 to 4 [25]; *K* is the crystallization rate constant, which usually indicates the speed of crystallization rate, and *Xv*(*t*) is the relative crystallinity corresponding to the crystallization time t. This section is divided by subheadings. It provides a concise and precise description of the experimental results, their interpretation, and the experimental conclusions that can be drawn.

The Jeziorny method describes the non-isothermal crystallization kinetics of polymers on the basis of the Avrami equation; by modifying the parameters of non-isothermal crystallization, the non-isothermal crystallization mechanism of polymers was determined.

**Figure 8:**
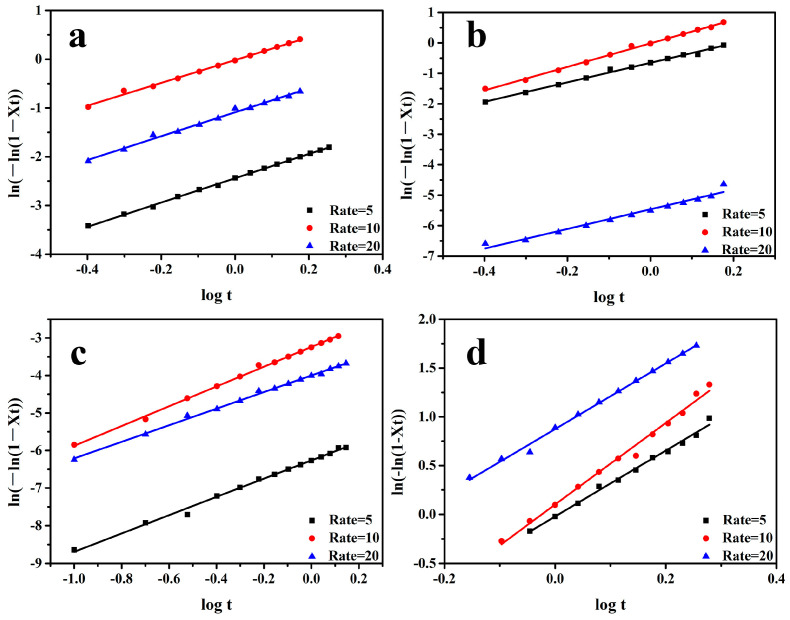
Non-isothermal crystallization kinetics curves of the PP/SCAB composite. (**a**) PP; (**b**) PP/SCAB; (**c**) PP/Znst; (**d**) PP/SCAB-Znst.

According to Equation (3), the curve of *log*[−*ln*(1 − *X_v_*(*t*))] to *log*(*t*) was obtained. From the slope, we obtained the Avrami exponent *n*, and log *K* was obtained from the intercept. For this *K*, the cooling rate of *K* was corrected using Equation (4), and the non-isothermal crystallization kinetics curves and parameters of the PP/SCAB composite are recorded in Figure 8 and Table 3.

It can be observed from Figure 6 and Figure 7 and Table 3 that *t*_1/2_ decreases with the cooling rate increases. This is because the samples require more time to nucleate at lower the cooling rates. As the cooling rate gradually increases, the nucleation time of the samples decreases, leading to an increase in the crystallization rate of PP.

It can be seen from Table 3 that the *n* value of PP and PP/Znst was about 2, which indicates that after the addition of Znst, the growth mode of PP did not change. However, the *n* value of the PP/SCAB and the PP/SCAB-Znst was about 3, which indicates that the addition of SCAB and SCAB-Znst, which can provide nucleation sites in the PP matrix, and the crystal growth mode of PP changed from two-dimensional growth to three-dimensional growth after adding SCAB and SCAB-Znst. Moreover, the log *K_c_* of the sample increased with the increase in cooling rate, while the semi-crystallization time (*t*_1/2_) decreased. The result showed that the crystallization rate of the PP/SCAB composites had a positive correlation to the cooling rate. At the lower cooling rate, the time for the PP/SCAB composite to produce a crystal nucleus was longer. However, as the cooling rate gradually increased, the time for the composite to generate crystal nuclei was shortened, which indicates that the nuclei can form at lower temperatures. For instance, the *t*_1/2_ of the PP/SCAB composite decreased by 3.5% (PP/Znst), 10.6% (PP/SCAB), and 21.2% (PP/SCAB-Znst) with a cooling rate of 10 °C /min, which indicates that SCAB plays a certain role in accelerating the crystallization rate of PP. Moreover, the PP/SCAB-Znst showed the lowest *t*_1/2_, which proves that the dispersion property of SCAB was greatly improved. Moreover, Znst in SCAB-Znst may play the role of internal lubrication in the PP matrix, which can speed up the molecular chain rearrangement and accelerate the crystallization rate of PP [26].

### 3.6. POM of the PP/SCAB Composite

**Figure 9:**
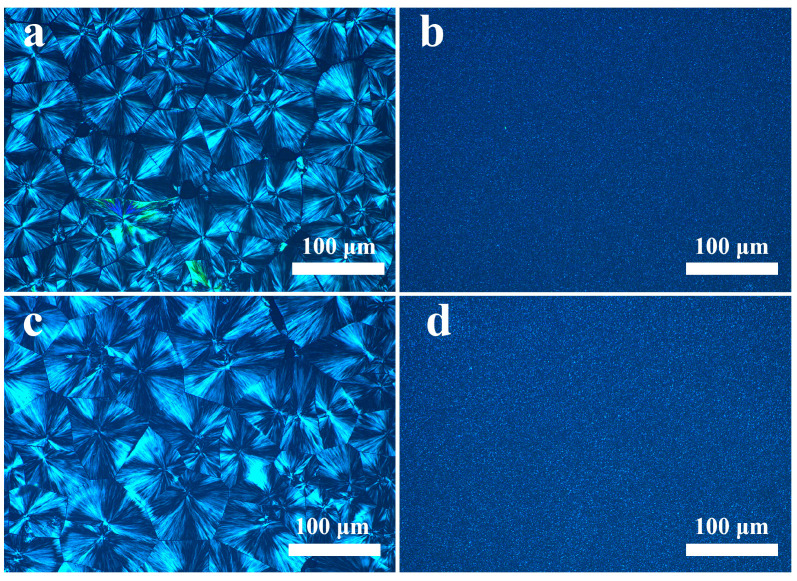
The POM morphology of the PP/SCAB composite at 135 °C. (**a**) PP; (**b**) PP/SCAB; (**c**) PP/Znst; (**d**) PP/SCAB-Znst.

The POM morphology of the PP/SCAB composite after isothermal crystallization at 135 °C is shown in Figure 9. It can be clearly seen that the spherical of pure PP (Figure 9a) and the PP/Znst (Figure 9c) exhibit an obvious black cross extinction phenomenon, and the particle sizes larger than 100 μm. As shown in Figure 9b, after the addition of SCAB, the spherulite was obviously refined, and the particle size was much smaller than 100 μm. The nuclei density of the PP/SCAB was greatly increased, the boundaries of the spherulite became blurred, and the homogeneity was improved. At the same observation scale, due to the fact that the addition of Znst can promote the dispersion of SCAB in the PP matrix, PP/SCAB-Znst has the smallest spherulite size. In general, the smaller size of the spherulite leads to the molecular chain bonding becoming tighter, and the degree of structural ordering becomes higher [27,28]. Therefore, the result is strong evidence of the improved crystallization properties of the PP/SCAB composite, and Znst improved dispersion of SCAB in the PP matrix.

### 3.7. Lubrication Mechanism of the PP/SCAB Composite

**Figure 10:**
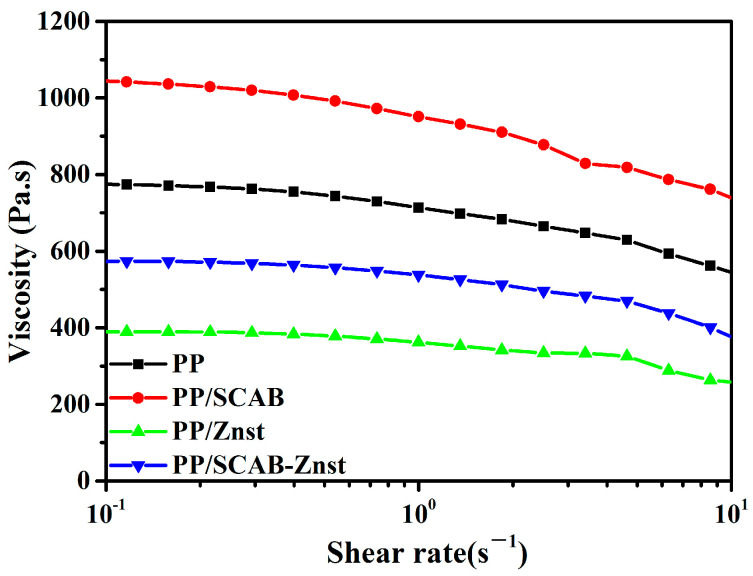
Relationship between viscosity and shear rate of the PP/SCAB composite at 200 °C.

The relationship between viscosity and shear rate of the PP/SCAB composite at 200 °C is shown in Figure 10. It can be seen from the figure that the viscosity of PP/SCAB was higher than that of PP. The main reason for this result may be the aggregation of SCAB in the PP matrix [29]. Moreover, PP/Znst had the lowest viscosity, and the viscosity of PP/SCAB-Znst was lower than that of PP at the same shear rate. The result indicated that Znst can promote the rearrangement of molecular chains in the direction of shear, which showed a great lubrication effect on PP [30,31].

The *Arrhenius* equation was used to further explore the Rheological properties of the PP/SCAB composite, as shown in Equation (5) [32]:

(5) $$\eta^{*}=\mathrm{Aexp}(\Delta E/RT),$$

where η* is the viscosity, Δ*E* is the viscous flow activation energy, *A* is the viscosity constant, and *R* is the gas constant (8.314 J/mol∙K).

Equation (5) is typically rearranged in a linear form as the logarithm Equation (6):

(6) $$Ln\eta^{*}=lnA+\frac{\Delta E}{R}\cdot \frac{1}{T}$$

**Figure 11:**
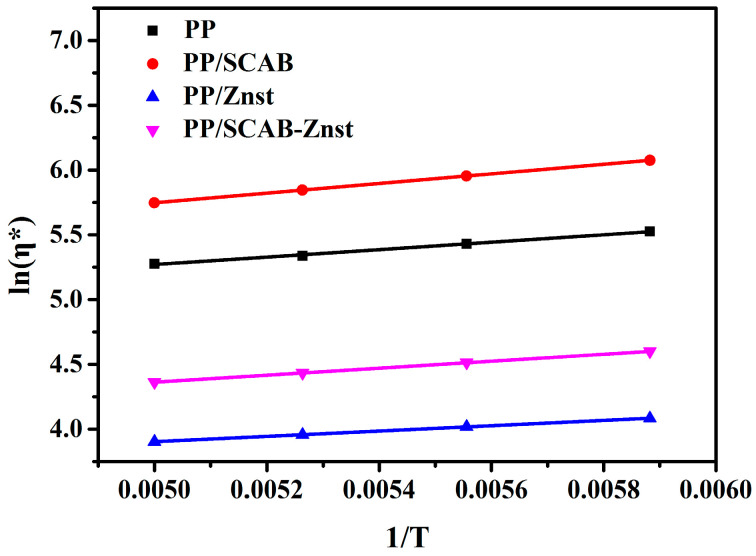
Relationship between viscosity and temperature of the PP/SCAB composite.

The curves and parameters obtained through Equation (6) are recorded in Figure 11 and Table 4, respectively. It can be seen from Figure 11 that the Lnη* shows a linear relationship with 1/T. The result shows that the melt flow behavior of the PP/SCAB composite is consistent with the *Arrhenius* equation. The addition of SCAB resulted in a significant increase in Lnη*, which demonstrates the aggregation phenomenon in the PP matrix. It is clear from Table 4 that the order of *lnA* and ΔE of the PP/SCAB composite is PP/Znst < PP/SCAB-Znst < PP < PP/SCAB. The result presents that Znst exhibits internal lubrication in PP [33]. Znst can be evenly dispersed between the molecular chains of PP, and the molecular chain shielding effect can effectively reduce the friction and reduce the interaction force of the PP molecular chains and improve the flexibility of the PP molecular chains, which can greatly improve the crystallization ability of PP [34].

### 3.8. Mechanical Properties of the PP/SCAB Composite

**Figure 12:**
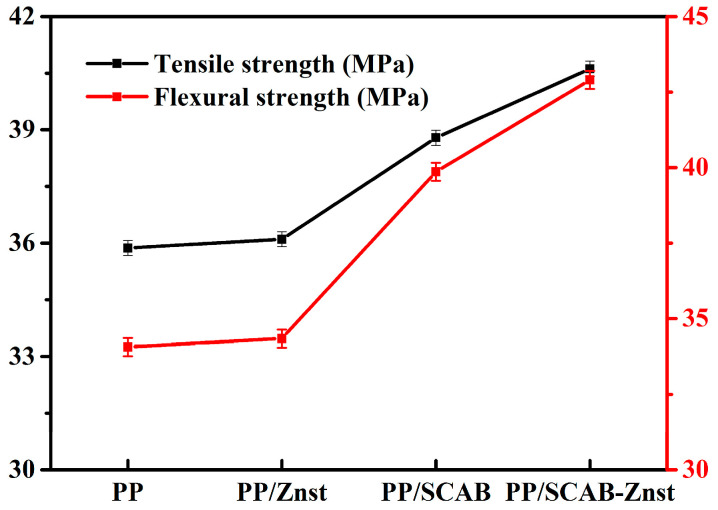
Mechanical properties of the PP/SCAB composite.

The mechanical properties of the PP/SCAB composite are shown in Figure 12. It can be seen from Figure 12 that the tensile strength and flexural strength of PP/Znst remain basically unchanged compared with pure PP. The result indicated that Znst alone had no effect on the crystalline structure of the PP/SCAB composite, and the contribution to the improvement of mechanical properties of the PP/SCAB composite was not significant.

The flexural strength of pure PP, PP/SCAB, and PP/SCAB-Znst were 35.8 MPa, 38.8 MPa, and 40.6 MPa, respectively. The results demonstrate that SCAB acts as a heterogeneous nucleation, which can make the large spherulites of PP become smaller spherulites or microcrystals. As the crystalline grain is refined, the crystal of the PP transitions from the micron to the nanometer, which is proven by the POM morphology of the PP/SCAB composite. The appearance of microcrystals also plays a self-reinforcing effect on the PP/SCAB composites, which was shown in the increase in flexural strength. In addition, SCAB-Znst contributes more to the increase in flexural strength of the composite, which indicates that the addition of Znst can significantly improve the dispersion of SCAB in PP [35]. The tensile strength of PP/SCAB and PP/ SCAB-Znst reached the value of 39.8 MPa and 42.9 MPa, respectively, compared with pure PP (34.1 MPa). The reason for this result is that SCAB acts as a heterogeneous nucleation, which can increase the *X_c_* of the PP/SCAB composite [36]. Moreover, the tensile strength of PP/SCAB-Znst reached its maximum value with the uniform dispersion of SCAB in the PP matrix.

### 3.9. Dispersion Property of SCAB in the PP Matrix

**Figure 13:**
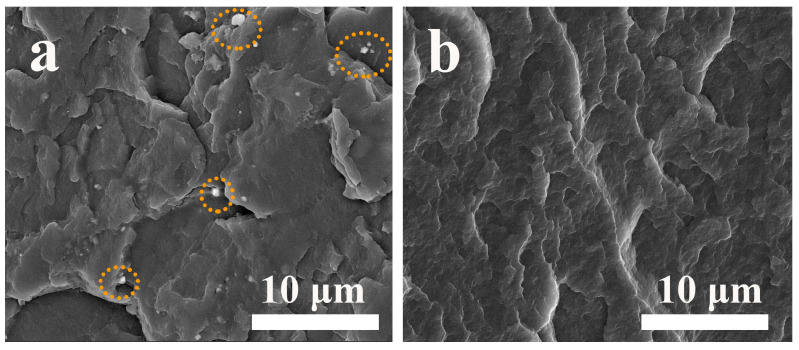
Fracture surface morphology of the PP/SCAB composite. (**a**) PP/SCAB; (**b**) PP/SCAB-Znst.

The fracture surface morphology of the PP/SCAB composite is shown in Figure 13. It can be seen from Figure 13a that the fracture surface of PP/SCAB is uneven and exhibits obvious post-yield fracture characteristics, which are typical of ductile fracture morphology [37]. Meanwhile, it can be clearly observed that white particles (SCAB) aggregate on the surface of the PP matrix, which may cause the PP/SCAB composite to easily separate and form defects when subjected to external forces that may pose a threat to the mechanical properties of the PP/SCAB composite [38,39]. However, it can be seen from Figure 13b that the addition of Znst can increase the dispersion property of SCAB in the PP matrix, which is manifested by the absence of obvious particles on the surface of the fracture surface of PP/SCAB-Znst [40]. Compared with PP/SCAB, the fracture surface of PP/SCAB-Znst is smoother, which leads to an increase in rigidity. This is strong evidence that PP/SCAB-Znst has the best mechanical properties.

## 4. Conclusions

In this work, in order to improve the dispersion of organic nucleating agents in the PP matrix and provide lubricating effects, SCAB was physically blended with Znst to obtain the SCAB-Znst composite nucleating agent. The PP/SCAB composite was prepared through melting blending. The FTIR and TG results indicated that Znst did not alter the chemical structure of SCAB. Moreover, the SCAB-Znst composite nucleating agent can be applied to the modification of PP. The SEM and the fracture surface morphology of the PP/SCAB composite illustrated that the addition of Znst greatly reduced the aggregation phenomenon of SCAB in the PP matrix. The results of the rotary rheometer indicated that Znst exhibits internal lubrication in PP and is evenly dispersed between the molecular chains of PP, which can effectively reduce the friction and reduce the interaction force of PP molecular chains and improve the flexibility of PP molecular chains. The DSC results illustrated that the crystallization properties of PP were improved. Compared with pure PP, the *T_c_* of the PP/SCAB composite increased by 13.48 °C, and the PP/SCAB-Znst composite increased by 14.96 °C, respectively. The increasing trend of *X_c_* is consistent with that of *T_c_*. The addition of SCAB can greatly improve the flexural strength and tensile strength of PP. Moreover, the flexural strength and tensile strength of PP further improved with the improvement of the dispersion of SCAB in the PP matrix. The results demonstrated that Znst can promote the dispersion of SCAB in the PP matrix while exerting a lubricating effect, which enabled the enhancement of the crystalline and mechanical properties of PP.

## Figures and Tables

**Table 1:** The formula of PP/SCAB composite.

Sample Name	PP (g)	SCAB (g)	Znst (g)
PP	200	-	-
PP/SCAB	200	0.6	-
PP/ZnSt	200	-	0.6
PP/SCAB-ZnSt	200	0.6	0.6

**Table 2:** Non-isothermal crystallization parameters of the PP/SCAB composite.

Sample	*T_c_* (°C)	*T_m_* (°C)	Δ*H_m_* (J/g)	*X_c_* (%)
PP	117.20	169.05	95.36	53.88
PP/SCAB	130.68	167.17	99.87	56.42
PP/Znst	117.76	169.91	95.43	53.92
PP/SCAB-Znst	132.16	167.06	99.91	56.44

**Table 3:** Non-isothermal crystallization kinetics parameters of the PP/SCAB composite.

Sample	*φ*	*T_c_* (°C)	Δ*H_c_* (J/g)	*n*	*log k_c_*	*t*_1/2_ (min)
PP	5	120.14	109.8	2.5	−2.44108	1.87
	10	117.20	109.7	2.3	−0.01653	1.13
	20	113.80	104.7	2.5	−1.08751	0.71
PP/SCAB	5	134.33	114.2	3.2	−0.65311	1.74
	10	130.68	111.3	3.8	−0.01444	1.01
	20	126.85	111.5	3.4	−0.46192	0.52
PP/ZnSt	5	120.27	109.8	2.4	−6.26352	1.82
	10	117.76	110.2	2.6	−3.23878	1.09
	20	115.00	104.9	2.2	−4.00041	0.68
PP/SCAB-ZnSt	5	135.49	115.8	3.4	−0.02088	1.71
	10	132.16	116.1	4.1	0.10153	0.89
	20	128.50	113.5	3.4	0.87485	0.45

**Table 4:** *Arrhenius* equation parameters of the PP/SCAB composite.

Samples	*lnA*	Δ*E*	*R* ^2^
PP	3.84	34.47	0.99825
PP/SCAB	3.89	44.68	0.99888
PP/Znst	2.88	24.62	0.99985
PP/SCAB-Znst	3.02	30.36	0.99996

## Data Availability

The original contributions presented in the study are included in the article, further inquiries can be directed to the corresponding authors.

## References

[1] Tadele D., Roy P., Defersha F., Misra M., Mohanty A.K. (2020). A Comparative Life-Cycle Assessment of Talc- and Biochar-Reinforced Composites for Lightweight Automotive Parts. Clean Technol. Environ. Policy.

[2] Pal S., Bhattacharjee P. (2018). Polypropylene-Based Packaging Materials for Shelf-Life Enhancement of Yellow Corn (*Zea mays*) Kernels: Effects on Lutein, Aflatoxin Content, Sensory, and Nutritional Profiles. J. Food Process. Preserv..

[3] Ghanbari A., Seyedin S., Haddadi S.A., Nofar M., Ameli A. (2021). Reinforcing Potential of Recycled Carbon Fibers in Compatibilized Polypropylene Composites. J. Polym. Res..

[4] Wang K., Chen L., Gao Y., Jiang D., Quan Y., Yan S. (2022). Effect of Morphology Development on the Low-Temperature Tensile Properties of PP/POE Blends. J. Appl. Polym. Sci..

[5] Ong M.Y., Chow W.S. (2020). Kinetics of Crystallization for Polypropylene/Polyethylene/Halloysite Nanotube Nanocomposites. J. Thermoplast. Compos. Mater..

[6] Huang C.-W., Yang T.-C., Hung K.-C., Xu J.-W., Wu J.-H. (2018). The Effect of Maleated Polypropylene on the Non-Isothermal Crystallization Kinetics of Wood Fiber-Reinforced Polypropylene Composites. Polymers.

[7] Wu B., Zheng X., Xu W., Ren Y., Leng H., Liang L., Zheng D., Chen J., Jiang H. (2023). β-Nucleated Polypropylene: Preparation, Nucleating Efficiency, Composite, and Future Prospects. Polymers.

[8] Deng Z., Meng X., Li C., Yao Z., Gong W. (2023). Effects of Halloysite Nanotubes Modified by Organic Phosphate on the Performance Improvement for Polypropylene. J. Appl. Polym. Sci..

[9] Liu Y., Wang D., Hu S., Zhou J., Li L., Huo H. (2019). Optimizing Nanoscale Morphology and Improving Carrier Transport of PCDTBT-PCBM Bulk Heterojunction by Cyclic Carboxylate Nucleating Agents. Org. Electron..

[10] Zhang Y.-F., He B., Hou H.-H., Guo L.-H. (2017). Isothermal Crystallization of Isotactic Polypropylene Nucleated with a Novel Aromatic Heterocyclic Phosphate Nucleating Agent. J. Macromol. Sci. Part B-Phys..

[11] Shan H., He J., Zhu B., Zhou J., Huo H. (2022). The Role of the Commercial Nucleating Agent HPN-68L in the Stretchable and Electrical Properties of Solvent Vapor Annealed P3HT. J. Mater. Chem. C.

[12] Zenzingerova S., Kudlacek M., Navratilova J., Gajzlerova L., Jaska D., Benicek L., Cermak R. (2023). The Competition between Self-Seeding and Specific Nucleation in Crystallization of Long-Chain Branched Polypropylene. Express Polym. Lett..

[13] Wang Z., Yang W., Liu G., Mueller A.J., Zhao Y., Dong X., Wang K., Wang D. (2017). Probing into the Epitaxial Crystallization of β Form Isotactic Polypropylene: From Experimental Observations to Molecular Mechanics Computation. J. Polym. Sci. PART B-Polym. Phys..

[14] Li X., JinRong Z., Yan L., YueFei Z. (2022). In Situ Synthesis of Calcium Pimelate as a Highly Dispersed Β-nucleating Agent for Improving the Crystallization Behavior and Mechanical Properties of Isotactic Polypropylene. Polym. Adv. Technol..

[15] Fuhua L., Mi Z., Shuangdan M., Jianjun Z., Kezhi W., Jun L., Xinde C., Bo W., Yinghui W. (2022). The Influence of Metal Lithium and Alkyl Chain in the Nucleating Agent Lauroyloxy-Substituted Aryl Aluminum Phosphate on the Crystallization and Optical Properties for iPP. Polymers.

[16] He X., Li Y., Nie M., Wang Q. (2016). Root-like Glass Fiber with Branched Fiber Prepared via Molecular Self-Assembly. RSC Adv..

[17] Zhao T.-J., Lin F.-H., Mao S., Dong Y.-P., Zhao J.-L., Cui W.-J., Wang S.-H., Ning D.-Y., Lu J.-Q., Wang B. (2024). Functionalization Modification of the Fischer-Tropsch Wax to Improve the Mechanicaland Crystallization Properties of the Recycled Polypropylene/Attapulgite Composites. Polymer.

[18] Mao S.-D., Zhang M., Lin F.-H., Li X.-Y., Zhao Y.-Y., Zhang Y.-L., Gao Y.-F., Luo J., Chen X.-D., Wang B. (2022). Attapulgite Structure Reset to Accelerate the Crystal Transformation of Isotactic Polybutene. Polymers.

[19] Gang W.U., Zhen-bin C., Yang-dong L.I.U., Si-yuan L.U. (2023). Effect of Stearate on Performance of Transparent Modified Polypropylene with Sorbitol-Based Nucleating Agent. Plast. Sci. Technol. Suliao Ke-Ji.

[20] Balkaev D., Neklyudov V., Starshinova V., Stolov M., Amirova L.M., Ziyatdinova A., Amirov R.R. (2021). Novel Nucleating Agents for Polypropylene and Modifier of Its Physical-Mechanical Properties. Mater. Today Commun..

[21] Dotson D.L., Spalding M.A., Chatterjee A.M. (2017). Nucleating Agents for Polyethylene. Handbook of Industrial Polyethylene and Technology.

[22] Turgut G., Isiksel E., Kahraman G., Eren T., Özkoç G. (2018). Synthesis of Phosphorus- and Phenyl-Based ROMP Polymers and Investigation of Their Effects on the Thermomechanical and Flammability Properties of a Polypropylene-IFR System. J. Appl. Polym. Sci..

[23] Wang B., Zhang H.-R., Huang C., Xiong L., Luo J., Chen X. (2017). Study on Non-Isothermal Crystallization Behavior of Isotactic Polypropylene/Bacterial Cellulose Composites. RSC Adv..

[24] Seven K., Cogen J., Gilchrist J. (2016). Nucleating Agents for High-Density Polyethylene-A Review. Polym. Eng. Sci..

[25] Gholami F., Pircheraghi G., Rashedi R., Sepahi A. (2019). Correlation between Isothermal Crystallization Properties and Slow Crack Growth Resistance of Polyethylene Pipe Materials. Polym. Test..

[26] Kaizuka M., Sato H., Ozaki Y., Sato H. (2024). Visualization of Recrystallization Induced by Ultraviolet Degradation of a Polypropylene Film Using Raman Imaging. Appl. Spectrosc..

[27] Tian M., Yang Y., He W., Li J., Qin S., Yu J. (2016). Preparation, Characterization and Application of Silica Nanoparticle Micro-Aggregates with Circular Structures. Chem. J. Chin. Univ.-Chin..

[28] Xu R.R., Du B.X., Xiao M., Li J., Liu H.L., Ran Z.Y., Xing J.W. (2021). Dielectric Properties Dependent on Crystalline Morphology of PP Film for HVDC Capacitors Application. Polymer.

[29] Lei X., Liang M., Zou H., Zhou S. (2023). A Holistic Evaluation of the Influence of Shear Rates and Matrix Viscosity on the Properties of Polypropylene/Multi-Walled Carbon Nanotubes Composites. Polym. Adv. Technol..

[30] Dai L., Wang X., Zhang J., Wang F., Ou R., Song Y. (2019). Effects of Lubricants on the Rheological and Mechanical Properties of Wood Flour/Polypropylene Composites. J. Appl. Polym. Sci..

[31] Martin-Alfonso J.E., Valencia C., Sanchez M.C., Franco J.M., Gallegos C. (2013). The Effect of Recycled Polymer Addition on the Thermorheological Behavior of Modified Lubricating Greases. Polym. Eng. Sci..

[32] Lugt P.M. (2023). On the Use of the Arrhenius Equation to Describe the Impact of Temperature on Grease Life. Tribol. Int..

[33] Li F.-J., Tan L.-C., Zhang S.-D., Zhu B. (2016). Compatibility, Steady and Dynamic Rheological Behaviors of Polylactide/Poly(Ethylene Glycol) Blends. J. Appl. Polym. Sci..

[34] Gong L., Yin B., Li L., Yang M. (2012). Morphology and Properties of PP/EPDM Binary Blends and PP/EPDM/Nano-CaCO_3_ Ternary Blends. J. Appl. Polym. Sci..

[35] Liu M., Chen K., Yu S., Zhang R., Jia M., Pan K., Xue P. (2022). Light-Weight and High-Strength PP/CaCO_3_ Composites by Die Drawing: Effect of Drawing Ratios. Polym. Eng. Sci..

[36] Han H., Hu S., Feng J., Gao H. (2011). Effect of Stearic Acid, Zinc Stearate Coating on the Properties of Synthetic Hydromagnesite. Appl. Surf. Sci..

[37] Bai H., Wang Y., Song B., Fan X., Zhou Z., Li Y. (2009). Nucleating Agent Induced Impact Fracture Behavior Change in PP/POE Blend. Polym. Bull..

[38] Burbano-Garcia C., Araya-Letelier G., Astroza R., Silva Y.F. (2022). Adobe Mixtures Reinforced with Fibrillated Polypropylene Fibers: Physical/Mechanical/Fracture/Durability Performance and Its Limits Due to Fiber Clustering. Constr. Build. Mater..

[39] Yousefi A.A., Rezaei M., Naderpour N. (2023). Hybrid Multiwalled-Carbon Nanotube/Nanosilica/Polypropylene Nanocomposites: Morphology, Rheology, and Mechanical Properties. Polym. Compos..

[40] Zhang Q., Li K., Fang Y., Guo Z., Yang X., Sheng K. (2022). Improvements in Compatibility and Properties of Biocomposites Modified through Nanosilica Attachment. Iran. Polym. J..

